# Achieving Dietary Sodium Recommendations and Atherosclerotic Cardiovascular Disease Prevention through Culinary Medicine Education

**DOI:** 10.3390/nu12123632

**Published:** 2020-11-26

**Authors:** Alexander C. Razavi, Amber Dyer, Matthew Jones, Alexander Sapin, Graciela Caraballo, Heather Nace, Kerri Dotson, Michael A. Razavi, Timothy S. Harlan

**Affiliations:** 1 Goldring Center for Culinary Medicine, Tulane University School of Medicine, New Orleans, LA 70119, USA; adyer4@tulane.edu (A.D.); mjones41@tulane.edu (M.J.); asapin@tulane.edu (A.S.); hnace@tulane.edu (H.N.); mrazavi@tulane.edu (M.A.R.); 2 Department of Epidemiology, Tulane University School of Public Health and Tropical Medicine, New Orleans, LA 70112, USA; 3 George Washington University Culinary Medicine Program, George Washington University School of Medicine and Health Sciences, Washington, DC 20052, USA; caraballo@gwmail.gwu.edu (G.C.); kdotson@email.gwu.edu (K.D.); drdrmrmd@mac.com (T.S.H.)

**Keywords:** dietary sodium, salt, medical education, nutrition, diet, cardiovascular diseases, hypertension, health literacy, minority health

## Abstract

Sodium-reduction initiatives have been a cornerstone of preventing hypertension and broader atherosclerotic cardiovascular disease (ASCVD) since the early 1970s. For nearly 50 years, public health and clinical guidelines have concentrated on consumer education, behavioral change, and, to a lesser extent, food policy to help reduce sodium intake among Americans. While these efforts undoubtedly helped improve awareness, average sodium consumption remains at approximately 4200 mg/day in men and 3000 mg/day in women, well above the United States Dietary Guidelines of 2300 mg/day. Culinary medicine is an emerging discipline in clinical and public-health education that provides healthcare professionals and community members with food-based knowledge and skills. With the hands-on teaching of kitchen education to individuals, culinary medicine provides eaters with tangible strategies for reducing sodium through home cooking. Here, we review opportunities for culinary medicine to help improve both individual- and population-level sodium-reduction outcomes through five main areas: increasing adherence to a plant-forward dietary pattern, food literacy, the enhancement of complementary flavors, disease-specific teaching-kitchen modules, and the delivery of culturally specific nutrition education. Through this process, we hope to further underline the value of formal, hands-on teaching-kitchen education among healthcare professionals and community members for ASCVD prevention.

## 1. Introduction

Sodium is a primary and ubiquitous constituent of our food supply, such that even determined individuals find it challenging to reduce their salt consumption. Excess sodium has a major role in the etiology and pathogenesis of hypertension [1], but also broader atherosclerotic cardiovascular disease (ASCVD) [2], directly contributing to increased arterial stiffness and adverse ventricular remodeling [3,4]. Overall, ASCVD continues to be the leading global cause of morbidity and mortality, as hypertension, coronary heart disease, stroke, and congestive heart failure affect 48% of the U.S. population, or approximately 121.5 million Americans [5]. Sodium-reduction initiatives in the United States date back to 1973 and 1979, when the American Heart Association and the American Medical Association advised citizens to aim for lower salt intake [6]. Over the course of nearly 50 years, medical and public-health professionals have aimed to reduce both individual- and population-level sodium consumption by enhancing consumer knowledge and food labeling, and to reform food policy. While all of these approaches improved awareness, Americans continue to consume nearly 1.5- to 2-fold higher-than-recommended daily intakes of sodium [7,8]. These observations demonstrate that our paradigm towards achieving dietary sodium recommendations may be incomplete, and suggest that more experiential nutrition-education techniques are required to help overcome an epidemic of sodium excess and improve primary ASCVD prevention. 

## 2. Culinary Medicine, Mediterranean Diet, and Sodium Reduction

Culinary medicine is an emerging concept in clinical and public-health education that provides healthcare professionals and community members with food-based knowledge and skills [9,10]. Culinary medicine is not defined or endorsed by a single dietary philosophy, and it may vary depending on a patient’s disease-specific dietary needs. However, curricula unquestionably focus on whole-food nutrition, while fostering knowledge among medical professionals about healthy dietary practices and counseling patients in need of dietary intervention about how to shift a majority of meals to being home-based. Several previous studies demonstrated that culinary-medicine interventions improved dietary-counseling competency for sodium reduction among medical students, residents, physicians, and nurses [11,12,13]. Furthermore, such interventions also led to increased adherence to Mediterranean diet practices among community members and healthcare professionals themselves. For example, compared to community members randomized to traditional nutrition counseling, those randomized to a 6 week hands-on teaching-kitchen education class reported a 1.5–2-fold higher consumption of vegetables and preparation of home-cooked meals [14], both of which are associated with an exceedingly lower quantity of sodium intake. Such initial data demonstrated a utility of teaching-kitchen education for sodium reduction, and suggest that the addition of culinary-medicine training should become a priority in medical schools and hospitals across the United States. 

The largest culinary-medicine programming platform in the U.S., the Health meets Food curriculum, has focused on translating Mediterranean diet nutrition principles for the American kitchen. Previous studies demonstrated that higher Mediterranean diet adherence is associated with a reduction in sodium intake and downstream ASCVD, including hypertension [15,16,17]. Health meets Food courseware has improved Mediterranean diet adherence across a wide variety of groups, including community members and their children, medical students, and physicians [11,12,13,14]. The curriculum teaches individuals to cook meals using recipes that target less than 500 mg of sodium per serving, which equates to a maximum of 1500 mg/day, leaving room to meet sodium guidelines with the addition of up to two snacks per day. For example, one of the first modules taught to medical students involves cooking a variety of spaghetti dishes that include different tomato sauces containing beef versus those containing higher ratios of vegetables and legumes. In addition to showing that the plant-based legume dish has less saturated fat compared to the dish with beef, cooking tomato sauce from scratch and/or choosing a tomato sauce that is low in sodium can drastically reduce mealtime salt intake while preserving flavor. Overall, teaching individuals the principles of a Mediterranean diet can reduce sodium intake through the consumption of a higher quantity of whole foods, vegetables, and plant proteins (tofu, beans, and legumes) that are traditionally prepared with lower sodium density compared to animal-based proteins [18,19]. Furthermore, the higher amount of potassium found in plant-forward dietary patterns, including the Mediterranean diet and Dietary Approaches to Stop Hypertension (DASH), can help preserve normotension and a low ASCVD risk, even in the setting of high–normal sodium intake [1,20].

## 3. Food Literacy

One of the primary goals of kitchen-based education is to improve food literacy. Food literacy describes a broad set of skills that are required to participate in healthy and responsible food- consumption behavior [21]. This means both possessing knowledge about healthy diet habits and incorporating knowledge into practice [21]. Food literacy in the setting of sodium reduction consists of knowing about the deleterious effects of sodium on health, understanding how to determine sodium content in foods, and practicing sodium-conscious dietary habits through such concepts as flavor balancing. Overall, food literacy is a promising avenue in the promotion of healthy dietary practices [22]. Several previous food-literacy scales have been proposed and created; however, no universal scales remain in place for measuring food literacy in communities [23]. Such scales were developed across several different demographic populations and included an overall similar category of knowledge domains (Table 1). To date, the Chinese Health Literacy Scale for Low Salt Consumption included the most domains related to sodium consumption and knowledge [24]. Although no standardized scale has been implemented, questions to assess the food literacy of class participants are incorporated within pre- and post-class Health meets Food surveys (medical trainees, medical professionals, and communities) that address attitudes, beliefs, and behaviors. Additionally, quizzes are given to medical trainees and professionals after the pre-class portion of the modules to determine the uptake of food-literacy concepts and knowledge.

Approximately one-quarter of all people in the United States do not have adequate food literacy [25]. Thus, a substantial proportion of Americans have problems interpreting nutrition labels and understanding how dietary behaviors affect chronic-disease development, which are large barriers to ASCVD prevention efforts. Individuals with higher food literacy and dietary-sodium awareness have lower sodium intake [26,27]. In patients with heart failure, those who have higher levels of food literacy have lower sodium consumption, improved control of their disease, and higher quality of life [28]. Knowledge about sodium and its health effects has a significant association with dietary practices and sodium consumption. Awareness of proper sodium-consumption regimens and knowledge of food-sodium content decrease sodium consumption [27]. Promoting food literacy with a warning label about sodium content in a food leads to decreased sodium consumption [8]. Improving food literacy through nutrition education improves knowledge about sodium intake and modifies consumption behavior. Teaching people how to read nutrition labels and identify food sodium content promotes improved self-regulation of sodium consumption [29,30].

## 4. Enhancement of Complementary Flavors

From a culinary perspective, salt has many desirable properties (e.g., ingredient preservation, curing, tenderizing, and flavor enhancement). The addition of salt to a dish enhances even the less palatable meals and is largely attributed to how “flavorful” a dish is. In our current environment, ultraprocessing techniques also add sodium to make food more addicting. Simply removing salt from a dish without balancing other flavors that uniformly activate the taste buds can lead to undesirable taste and a lack of adherence to a low-sodium diet. To successfully navigate the removal of salt from a dish, consideration must be given to the properties that salt provides and the sensory perception that accompanies salt ingestion [6].

Analyzing taste and flavor can appear to be referring to the same thing. However, at a closer look, flavor is composed of the different sectors of taste. The definition of the word “taste” once was singular and originated with an individual’s perception or connection with what constitutes the five basic tastes: sweet, salty, sour, bitter, and umami [31]. Sensory receptors on the tongue signal perceived taste to the brain’s afferent sensory neurons, and these neurons’ fibers travel along cranial nerves VII, IX, and X to reach their first synapse at the nucleus of the solitary tract in the medulla [31]. The second meaning of “taste” is part of the definition of flavor—the multisensory experience of smell, texture and touch, and sight and sound that integrates aroma, mind, body, and spirit into the experience of eating food. Learning how to recognize various tastes and the foods that are composed of these five basic tastes promotes skillful culinary techniques and elevates the patient’s experience with food. After understanding the basic tastes, attention to the skill of combining or manipulating the foods in which these tastes naturally occur produces complex and complementary flavors. An essential aspect of great cooking is harmonious flavor building. This commonly involves utilizing more spices, herbs, flavor-enhancing cooking techniques (e.g., charring, caramelizing, searing, and sauteing), and other flavorings that can best accentuate particular ingredients to enhance a dish.

The addition of certain ingredients with a higher threshold of flavor complexity can impact the cooking or manufacturing process of foods, which may assist in reducing the need for added sodium (Table 2). For example, the addition of herbs, spices, citrus, vinegars, and condiments that impart distinctive tastes is used in replacement of or in conjunction with salt when creating lower-sodium meals at home [6]. The combination of foods with a higher-sodium content like those mentioned can enhance the sodium-level perception when paired with other foods that are naturally low in sodium (e.g., fresh fruit, vegetables, grains, and legumes) in dishes or meals in a way that meets an individual’s satiety of the meal within the recommended daily intake of sodium. For example, stir-fry ingredients have characteristics that work together to enhance and balance the flavor of that dish. The fundamental flavors of a stir-fry sauce marry to provide the tastes of salt and umami (soy sauce), heat and spice (peppers), tang and sourness (rice vinegar or citrus juice), and sweetness (bell peppers, honey, or pineapple). These ingredients bring a presence of compatibility, but the dish does not feel simple when ingested. The dish is perceived as well-rounded, complex, and composed of notes of various taste receptors. Using these ingredients together is the way to achieve this harmony of complexity and reduce sodium content in meals [32].

The Health meets Food curriculum offers hands-on cooking classes either in person at a teaching kitchen or virtually. The purpose of these classes is to teach medical trainees and professionals how to cook and better counsel their patients in healthy-lifestyle modifications to reduce risk factors of chronic diseases in individuals and communities. All Health meets Food recipes must meet the following nutritional guidelines (Table 3): a caloric goal per meal of 300–400 for breakfast, 400–500 for lunch, and 500–600 for dinner; at least 5–10 grams of fiber per meal; no more than 500 milligrams of sodium per meal (less than 300 milligrams per meal for low-sodium patients); at least 5 grams of protein per meal; less than 3 grams of saturated fat per meal; and emphasis on plant-based fats and no trans fats. These guidelines help to provide a dietary-pattern framework that is healthful and achievable for the average American.

## 5. Disease-Specific Modules for Sodium Reduction

Health meets Food offers a variety of condition- and disease-specific modules that go even farther to highlight sodium-restricted flavorful cooking. These modules focus on hypertension, coronary heart disease, and congestive heart failure. One vital module provides more in-depth analysis, discussion, and reconceptualization of approaches in caring for hypertensive patients and combatting a sodium-restricted diet: Health meets Food Condition- and Disease-Specific Module 6: Renal Physiology, Hypertension, Sodium and Potassium Homeostasis, Sodium Reduction, and Flavor Building. This hands-on cooking module teaches clinicians achievable, informative, and real-world applications for discussing the effects of high-sodium diets with a patient. The module’s pre-class quiz, study materials, readings, lectures, and in-class exercises are provided in a traditional education method. The module provides an understanding of the similarities and differences of the Mediterranean diet and DASH, discusses hidden sources of sodium, and calculates and compares a patient’s recommended dietary allowance (RDA) of macronutrients and micronutrients. The module also covers competencies of patient dietary counseling recommendations, including food substitutions, on-the-go food options, and at-home cooking modifications. The significance of blood-pressure readings, the potential implications of variances in blood-pressure results, and the general causes of hypertension other than excessive dietary-sodium intake are all important concepts. The cooking portion of the module provides hands-on practice of culinary, clinical, and nutritional objectives, including ingredient replacements/substitutions, sodium reduction with cooking, and strategic flavor building. The purpose, functionality, and desirability of salt are discussed when cooking, and they are examined to understand and best balance flavors without adding excess table salt. The class menu offerings are all sodium-restricted meals that meet the Health meets Food nutritional guidelines. The class menu was designed to emphasize the utilization of various cooking techniques to build complementary and complex flavors that generate a low-sodium meal that is pleasurable for patients who have not adapted to a low-sodium lifestyle.

## 6. Minority Populations and Culturally Sensitive Nutrition Education

While sodium-reduction initiatives are necessary across the entire U.S. population, they are especially vital within minority populations who are disproportionately affected by hypertension and ASCVD when compared to White populations. When compared by sex and race in 2013, non-Hispanic Black men had the highest age-adjusted mortality attributable to ASCVD, followed by non-Hispanic White men and, lastly Hispanic men [33]. Overall, in 2013, women had a lower incidence of age-adjusted mortality attributable to ASCVD than men, but followed the same patterns as men when compared by race, with non-Hispanic Black women most affected, followed by non-Hispanic White women and lastly Hispanic women [33]. This disparity can be explained in part by the high burden of traditional ASCVD risk factors among ethnic and racial minorities, including a higher prevalence of hypertension, obesity, diabetes mellitus, and physical inactivity [34,35,36]. Although Hispanic adults have a lower incidence of mortality due to ASCVD than that of non-Hispanic Black and White adults, the burden of ASCVD risk factors is still quite high in this group [37].

In addition to social determinants of health and women’s health-specific factors such as menopausal status, sodium intake is a particularly important and targetable mediator of ASCVD risk factors, but data show that less than one-quarter of Hispanic and Black Americans meet the recommendations for daily sodium intake [38,39]. Previous data demonstrated that the engagement in healthy-lifestyle behaviors may differ according to both sex and race. Minority women in particular appear to be a high-risk population, with Black and Hispanic women being less likely to report consuming a healthy diet and engaging in physical activity compared to White women [37]. These disparities are in part due to inadequate access to healthy food options and outdoor spaces, and collective action is needed to address the social determinants of health to help improve cardiovascular health among minority populations. Furthermore, such data highlight the need for culturally specific sodium-reduction interventions within minority populations. 

Delivering culturally sensitive interventions through culinary-medicine classes may be a unique and effective approach to achieve sodium recommendations, improve primary prevention, and help close the gaps in racial health disparities. Preventive medicine is vital within minority populations, as they are more likely to lack health insurance or to be underinsured when compared with White populations [40]. Hands-on culinary-medicine classes are adaptable across a variety of cultures and can help address these issues by promoting healthy eating behaviors, such as reducing the quantity of salt in a meal. Recently, the Health meets Food community courseware was adapted for Hispanic populations and was launched in Spanish. The courseware was culturally tailored to maximize the success of participants, and included approachable, affordable recipes featuring traditional Hispanic foods and ingredients that are predominant in the Hispanic diet. By better engaging diverse populations in culturally specific interventions, culinary-medicine education has the potential to improve ASCVD prevention and reduce health inequities within underserved populations.

## 7. Conclusions

Here, we summarized key concepts and approaches to achieve sodium-restriction recommendations for cardiovascular health through culinary medicine. We specifically reviewed opportunities for culinary medicine to help improve both individual- and population-level sodium-reduction outcomes through five main areas, namely, increasing adherence to a plant-forward dietary pattern, food literacy, the enhancement of complementary flavors, disease-specific teaching-kitchen modules, and the delivery of culturally specific nutrition education. Overall, hands-on teaching-kitchen education may provide healthcare professionals and community members with a multidimensional approach to achieving sodium guidelines for ASCVD prevention.

## Figures and Tables

**Table 1:** Common food-literacy domains.

Ability to understand text Nutrition and health Energy sources in foods Household food measurement Food label and numeracy Food groups Consumer skills Engagement in dietary habits Taking a critical stance towards nutrition claims and their sources Calculating and converting sizes Influence of endorsement logos Knowledge of salt content Knowledge of diseases related to high salt intake Knowledge of international standards Myths about salt intake Attitudes towards salt intake Sodium food-consumption practices

**Table 2:** Balancing complementary flavors in cooking.

Sweet	Salty	Bitter	Sour/Acidic	Umami
Brings balance and roundness to a dish by balancing acidity and bitterness, and highlighting other flavors.	Aim to reduce and/or eliminate the consumption of such foods. Foods that do not appear to be salty can still have naturally high levels or added sodium in them.	Balances sweetness and cuts richness—best used as background flavor.	Brings brightness and adds an acidic flavor that balances sweetness.	Makes a dish savory or tasting of meat and gives depth of flavor.
Fruit juices, nectars, concentrates, reductions, caramelized onions, carrots, sweet potatoes, butternut squash, roasted peppers, honey, maple syrup, molasses, dried fruits, tomato paste, beets, reduced vinegars, wine	Table salt, cured meats, deli meat, bacon, processed foods, fried foods, French fries, cheese, breads and rolls, pizza, seasoning spice blends with salt, chicken or beef stock, olives, ketchup, cakes and pies, seaweed	Greens (kale, chard, dandelion, chicory, watercress, arugula) broccoli rabe, broccoli, cabbage, brussels sprouts, asparagus, some mustards, grapefruit, citrus rind/zest, beer, wine, teas (black and green)	Lemon, lime, orange, and pineapple juice, vinegars, wine (especially white), tamarind, pickled foods, cranberries, sour cherries, tomato products, citric acid	Tomato products (especially canned, such as paste), mushrooms (especially dried), cured or brined foods (olives), fish sauce, fermented foods (miso, fermented black beans, sauerkraut), liquid amino acids, seafood (especially dried), Worcestershire sauce, anchovies

Sourced in part from the American Heart Association Sodium-Reduction Initiative.

**Table 3:** Goals for balanced nutrition in Health meets Food recipes.

	Dinner Recipes
Calories	<600 kcal
Saturated Fat	<5 g
Sodium	<500 mg *
Carbohydrates	<50 g
Fiber	5–10 g
Sugar	<15 g

* Less than 300 mg/meal for high-risk patients, including those with hypertension and/or heart failure.
